# Observations on Italian Medical Institutions and Practice; a Brief Comparative Sketch of Some Points of French and English Practice, and Remarks on Italian Climate

**Published:** 1831-11

**Authors:** Edwin Lee

**Affiliations:** Member of the Royal College of Surgeons, and formerly House Surgeon to St. George's Hospital.


### ITALIAN MEDICAL INSTITUTIONS AND PRACTICE.

*Observations on Italian Medical Institutions and Practice; a brief Comparative Sketch of some Points of French and English Practice, and Remarks on Italian Climate.* By Edwin Lee, Esq., Member of the Royal College of Surgeons, and formerly House Surgeon to St. George's Hospital.

(Continued from page 312.)

The majority of the medical institutions of Italy are regulated in their internal management on the same principles as in France: the funds for their support are chiefly derived from government; the election of medical officers is decided by "concours;" patients are attended on by "sœurs de la charité, or infirmiers;" and the bodies of those who die are examined; the medical visits are made daily, and at an early hour in the morning; clinical lectures are delivered to students.

The medical profession is divided, as in France, into physicians, surgeons, obstetric practitioners, and pharmaciens. The division between medicine and surgery is more arbitrary than in France: in some parts of Italy the duties of the surgeon are confined to rendering manual assistance, and the performance of operations, while the physician prescribes for any constitutional disorder attendant on a surgical case. The principles by which the practice is guided resemble those adopted in France, and are generally based upon the Broussaian doctrine; the number of followers of the doctrine of Rasori and Tommassini is much diminished. The practice of exhibiting large doses of antimony, to supersede venesection in acute disease, is not frequently followed. The abstraction of blood from the system in small quantities is a mean pretty generally adopted: counter-irritation, by blisters or the tartar emetic ointment, is frequently employed; purgatives are seldom used, in comparison with the frequency of their employment in this country; sedatives, as hyoscyamus, digitalis, &c. are employed in many cases; tonics and stimulants rarely made use of.

In surgical cases, little or no medicine is ordered. Patients who die after accidents or operations, usually succumb under internal inflammations, which a better regulated after-treatment would in many instances prevent or subdue. Purulent deposits in the lungs and liver are of frequent occurrence in those who die after accidents or operations: the general temperance of Italians, however, and the purer air of their cities, render them less liable to the severe inflammatory attacks, and the high degree of constitutional irritation, which so frequently supervene on accidents or operations in this country.

### FLORENCE.

Florence, situated in a plain surrounded by the Appennines, has a population of 80,000 persons, and contains four hospitals: these are placed under the direction of a superintendent, appointed by the grand duke. The Spedale Santa Maria Nuova, for the reception of accidents, acute and chronic diseases, is a handsome edifice, and contains nearly 1000 beds; the bedsteads are made of iron, and have curtains. In some wards the beds are too closely placed together to allow a free circulation of air. The wards are clean, lofty, and well ventilated. A paper, stating the material circumstances and daily progress of each case is placed at the head of the bed. Patients are admitted on application, and are attended upon by "sœurs de la charité:" the bodies of those who die are invariably examined, and supply the dissecting room. There are two commodious operating theatres adjoining the surgical wards, one on the ground floor, the other on the first floor; a theatre for the delivery of anatomical and other lectures, a dissecting room, and a cabinet of pathological anatomy. Separate wards are appropriated to syphilitic patients, and patients afflicted with diseases of the eyes are placed in a darkened ward; a ward is also set apart for those patients who contribute towards their own maintenance, from three to five *pauls* a day.

Two professors of medical clinique, two of surgical clinique, and several assistant physicians and surgeons, who receive salaries, are attached to the service of this hospital. One half of these attendants do duty for six months of the year, at the expiration of which period they are replaced by the other half. The surgical visit takes place at eight o'clock every morning, and the medical visit at ten; the professors hold clinical discourses with pupils at the bedside of patients.

The lectures of the Faculties of Medicine and Surgery are delivered in this hospital, on the different branches of medical education; the examination of candidates for the diploma of medicine or surgery takes place before the members of the colleges. Medical candidates are previously obliged to take their degree at the university of Pisa.

The *Spedale di Bonifacio* is divided into two parts, one for insane patients, and the other for military patients, and those afflicted with incurable diseases. The hospital contains about 800 beds; the wards are spacious, airy, and particularly clean.

The average number of insane in the hospital is from 280 to 300: males are in greater proportion than females. Small cells, having each a window with iron bars, and containing a bed, are situate on either side of passages about fifty feet in length; each patient has a separate cell, but in the daytime they are indiscriminately allowed to walk about the passages, or in the open air. The patients are all clothed alike, in a white woollen dress. New patients are kept in separate rooms for a few days, in order that the peculiaries of their insanity may be observed. The greatest attention is paid to cleanliness throughout the establishment.

When confinement of the hands is necessary, a wooden case (*manchôt*) is used, into which both hands are placed, and kept bound to the abdomen by means of a strap passing round the waist. Furious patients are confined to a small darkened room, with well padded walls; the darkness is found to render the greater number of such patients tractable. Dr. Bruni is chief physician, and he conducts the treatment chiefly on moral principles; many of the patients are employed in mechanical occupations, gardening; the women in knitting, spinning, &c. The system formerly in use of employing depletory measures indiscriminately every summer, is now discontinued, and bleeding is employed only when there exists great exaltation of the cerebral functions.

The *Spedale degl' Innocente* is a large building for the reception of lying-in women and of infants; the number of which averages upwards of 2000 annually. The manner in which infants are bound up in swaddling cloths, somewhat resembling an Egyptian mummy, occasions frequent distortions of the limbs, and is productive of other bad effects.

The ergot of rye is administered at this hospital in those cases of protracted labour dependent on deficient contraction of the uterus.

There is at Florence a fourth hospital, containing about forty beds, for patients with acute and chronic diseases; also a Casa dei Poveri, or workhouse, on an extensive scale; and obsteric institutions in different parts of the city, for affording assistance at the habitations of poor women.

The *Societa della Misericordia.* This charitable establishment was instituted in the beginning of the 15th century, and counts among its members several of the nobility: its object is to render assistance to the sick poor, for whom the members perform many kind offices, and supply those who are treated at their own houses with necessaries of all kinds. They undertake the burial of the bodies of poor persons, and, in case of accident or disease, repair to the place where their services are required, and convey the patient to an hospital, or to their own residence. The brethren meet in a building in the Piazza del Duomo, where the affairs of the society are conducted by a committee: one or more of the committee is always in attendance at the central institution, to indicate to those on duty the place where their services are required. The sick are carried in covered litters, on the shoulders of the brethren, who maintain a profound silence, and are clothed from head to foot in a black domino, in order to conceal the persons of those who are thus engaged: ten, twelve, and sometimes more of the brethren, accompany each litter, and frequently relieve each other in supporting the burden.

In the Museum of Natural History, perhaps the finest existing, is the splendid collection of anatomical wax models, coloured according to nature, and exhibiting all the parts of the body, both conjointly and in detail, of the natural size. A room is allotted to each division of anatomy, as osteology, myology, &c. In addition to the anatomical models, are others illustrating the progress of utero-gestation, growth of the fœtus, &c. The models are in general pretty correct.

The most prevalent diseases at Florence are acute and subacute inflammation of the lungs, pleurisy, bronchial affections, dysentery, gastric irritation, rheumatism, and diseases of the eyes.

*State of Medicine.* No particular system in the treatment of disease is followed; the practice, however, leans to the Broussaian: all irritants and tonics are avoided; bleeding is very generally employed, small quantities of blood, as four, six, or eight ounces being abstracted at a time; consequently the frequent repetition is necessary, which has the effect, in many cases, of debilitating the patient, without effectually arresting acute inflammation. Hence one cause of the fatality attending acute inflammation of the lungs, known by the name of Mal di Petto, which so frequently occurs from the variable temperature of Florence. Leeches are often used, but not so generally resorted to as in France. Counter-irritation, by means of the application of the ointment of tartarised antimony, is frequently employed. Purgatives are seldom used, from a dread of their inducing gastro-enterite; sedatives are not unfrequently employed. Prussic acid, or the aqua lauro cerasi, is sometimes given in bronchial complaints.

Intermittents are not of frequent occurrence; they are treated by venesection when required, and by the exhibition of the preparations of bark. In continued fever, small general bleedings are employed; more frequently, however, the application of leeches to the pit of the stomach: lemonade and cooling drinks are resorted to.

Rheumatism is treated by bleeding, warm bath, and diaphoretics: the colchicum is not used; its effects in this class of diseases do not appear to be known.

In gastric and intestinal irritation, the application of leeches, and the administration of demulcent mixtures, are chiefly depended on.

No operation is performed without a previous consultation, at which the superintendent of hospitals is present. All persons, professional or not, are allowed to be present at operations, which are frequently performed by the more advanced pupils, under the guidance of the professor of surgery. Operations are in general pretty successful; patients are bled subsequent to their performance in all cases.

The lateral operation is performed for stone in the bladder; the bistoire caché is generally used for incising the neck of the bladder.

Hydrocele is treated by the operation of excision, the method by injection is adopted only in recent cases. The operation of couching is preferred in cataract.

Cases of strangulated hernia are operated on immediately: no other remedial means are employed, nor are any attempts made to effect reduction by the taxis. Strictures of the urethra are treated by confining the patient to bed, and passing an elastic gum catheter into the bladder, the size being gradually increased: when a catheter cannot be introduced into the bladder, a catgut bougie is passed into the urethra as far as possible, and retained against the stricture for some time: on withdrawing this, a catheter can generally be passed.

Fractures of the lower extremity are treated by placing the limb in the extended position: in fracture of the thigh, the limb is confined between two long splints, connected together by a piece of cloth, passed under the limb, and extending its whole length; the splints are tied together by pieces of tape. By this mode sufficient extension is not kept up, and shortening of the limb, more or less, is the consequence.

Ophthalmiæ, whether acute, chronic, or strumous, are treated by general or local depletion, warm emollient applications and fomentations; the patients being kept in a darkened room, stimulating collyria are scarcely ever had recourse to. Blisters are also seldom used in the treatment of diseases of the eyes. Under this treatment patients are long in recovering, and, from the debilitated state in which the organ is left, suffer frequent relapses after exposure to the sharp winds so prevalent in Florence.

Inflammations of joints are treated by rest, local abstraction of blood, and emollient cataplasms; counter-irritation is seldom employed. It is generally a long while before recovery takes place.

Mercury, both used externally and given internally, is chiefly trusted to for the cure of syphilitic complaints; its use, however, is not carried so far as to induce copious salivation.

Union by the first intention is generally attempted after operations, or recent wounds.

Abscesses are opened by a very minute aperture being made, and the matter forcibly pressed out. In two cases which I saw, this gave rise to severe inflammation of the part, accompanied with a high degree of febrile irritation.

I here subjoin the abstract of a few cases treated at the Spedale Santa Maria, as illustrative of the practice pursued.

*Case of supposed Spinal Disease.* December 10th, 1830. A girl, æt. seventeen, of good general health, but subject to irregular menstruation, on descending into a close cellar, fainted, and fell to the ground, three months ago: in falling, she struck her neck below the left ear, against some projecting body; abscess formed in this situation, was opened, and continued discharging for six weeks; at the expiration of which time it healed. Some days before her admission to the hospital, she lost the use of her left arm; shortly after, that of the left leg; and the right arm and leg subsequently became paralytic, in which state she was conveyed to the hospital, in the beginning of November. The functions of respiration, digestion, &c. continued unimpaired; as did those of the detrusor urinæ and sphincter ani. The case was considered as inflammation of the spinal marrow: bleeding, the repeated application of leeches, and blisters along the spine, the exhibition of strychnine, restricting the patient to low diet, and the formation of a sore by caustic in the situation of the previous abscess produced no amelioration.

A fortnight ago, she suddenly heard of the death of a near relation, and from that time constant movements of the limbs succeeded to the state of paralysis in which she had previously lain, and have continued ever since; the arms are constantly beating against the breasts, and the thighs and legs alternately flexed and extended with violence. Although pale, her countenance does not indicate the existence of organic disease; the intellectual and natural functions are not impaired; she answers questions readily; the tongue is clean; the urine of natural appearance, and slightly acid; skin cool, pulse weak; the sensibility of the skin is lessened, and she does not feel when pinched. The prognosis delivered by her physician is unfavorable; she takes no medicine, but leeches are occasionally applied along the spine.

I ascertained that the motions of the limbs, although pretty constant during the day, ceased altogether at night, and did not prevent her sleeping; they were also more violent when any one approached her bed, or conversed with her; but when she was not conscious of being observed, their violence was much lessened, and occasionally ceased altogether for a few seconds. She could put her hand to her head, or point to any thing she wanted. From a consideration of the peculiarities of the case, and of its long duration, I was led to suppose that, although the paralysis, in the first instance, might have been occasioned by some irritation of the nerves, consequent on the healing of the abscess, the present symptoms did not indicate an affection of the spinal marrow; that the complaint was essentially nervous, having great analogy with chorea, and, as in that disease, the motions of the limbs were, in some degree, kept up by habit. This view of the case I communicated to her physician, Professor Nespoli, who did me the honour to ask my opinion.

24th December. The depletory means have been discontinued, and the quantity of her food increased: has had several hysterical symptoms, as tremulous motions of the eyelids, loss of voice for a day or two, occasional fits of laughter; the movements of the limbs are less violent, and at times altogether cease; she sleeps well, and her appetite is good.

30th. The patient has been allowed a more full diet, and is improved in appearance; motions are now almost entirely confined to the hands, and cease if her attention can be diverted from her complaints; has occasional hysterical fits of laughter. From this time she gradually recovered: the motions of the hands ceased entirely; she regained flesh, and was discharged in the course of the month of January.

*Gangrenous Erysipelas of Thigh.* December 20th. A man, æt. twenty, of cachectic habit, after exposure to wet, was attacked seven days ago by fever preceded by rigors; two days after, the fever recurred, accompanied by pain and swelling of the lower part of the thigh, which gradually increased until his admission to the hospital on the 18th December, when he was bled to ℥vi., with temporary relief. The lower third of the thigh, and the knee, are much swollen, tender to the touch, and of a dark red hue; skin is hot; pulse 110, small; tongue coated; bowels confined. Ordered bleeding to ℥viii.; leeches to the affected part; and a nitrated drink, low diet; a laxative in the evening.

22d. Application of leeches was yesterday repeated. Patient has had several rigors; countenance is anxious; pulse quick and weak; skin cool; knee and thigh less painful, but retaining the dark hue, which is circumscribed, half-way up the thigh, by a distinct line of demarcation. The nitrated beverage repeated, and poultice applied to the thigh.

23d. The patient is scarcely sensible; breathes with difficulty; pulse quick and feeble. Bleeding to ℥vi.; leeches to the limb and poultice; medicine continued.

25th, died. On examination of the thigh, a quantity of pus was found surrounding the bone; periosteum thickened; the cellular texture above the knee in a sloughy state. No disease of viscera.

This is a case in which early and free incisions would have been made in this country, and, conjoined to an opposite mode of medical treatment, might have led to a different result.

*Sloughing Ulcer of the Leg.* A man, æt. sixty-two, was admitted into the hospital, with foul ulcer over the tibia: the patient was labouring under considerable nervous irritation; dry tongue, frequent pulse, skin warm. He was bled to ℥vi., and ordered to take lemonade.

On fourth day from his admission, the skin was cool; pulse weak and quick; tongue brown and dry; and he had slight delirium. Bleeding to ℥iii.; lemonade; poultice to the ulcer.

Sixth day. Wandered more in his talk; countenance sunk; pulse feeble; considerable difficulty in breathing:, bleeding to ℥iv.; lemonade. Eighth day, died.

*Strangulated Femoral Hernia.* A woman, æt. forty-five, was admitted under the care of Professor ANDREINI, with femoral hernia: symptoms of strangulated intestine had existed eleven hours. The patient had vomited several times, and had pain and tenderness on pressure of the lower part of her abdomen; the tumor was of the size of a walnut, and somewhat painful. Venesectio ad ℥viii. Two hours after admission, the operation was performed. No attempt was made to reduce the tumor by the taxis.

The patient being laid her whole length on the operating table, to which her legs were tied down, one of the pupils, under the direction of the Professor, made the preliminary incisions through the skin, fasciæ, and hernial sac, by which a knuckle of intestine, of a dark red colour, was exposed; the Professor then endeavoured to pass his finger up to the seat of stricture, but experienced considerable difficulty, which was doubtless increased by the tension in which the parts were kept, from the the position of the patient: having at length reached the stricture, a small straight bistourie caché was introduced by the Professor, and its division effected by an incision made in the direction of the symphysis of the pubis. The intestine was then reduced without difficulty.

Shortly after the operation, the patient had an evacuation from the bowels. In the course of eight hours, the pulse increased in frequency, accompanied with more tenderness of the abdomen on pressure. Eight ounces of blood were abstracted, by which the symptoms were for a time relieved; but, on the following day, the patient had pain all over the abdomen, attended with tension, vomiting, and quick small pulse. Venæsectio to ℥iij., and fomentations to the abdomen were employed. These means afforded no relief; hiccough supervened, and fœcal matter made its appearance at the wound; half an ounce of castor oil was then ordered, which occasioned slight evacuation per anum. The patient, however, died on the fourth morning from the operation.

In this case are chiefly remarkable the absence of urgent symptoms at the time of the patient's admission; the position in which she was placed for the operation; the nugatory effect of the means employed to combat the subsequent inflammation; and the non-employment of laxatives, from a fear of thereby increasing the inflammation of the bowels.

*Treatment after Lithotomy.* A boy, æt. eight years, underwent the lateral operation for stone in the bladder, and went on well until the fifth day from the operation, when he was attacked with pain and tenderness on pressure of the abdomen, accompanied by fever and constipation of the bowels; the urine passed freely away by the wound. He was ordered venesection to ℥vi., a poultice to the abdomen, and to drink lemonade. The severity of the symptoms was by these means mitigated, but the bowels continued constipated.

On the fifth day, the bleeding was repeated to the amount of ℥iv. On the seventh day, the same quantity of blood was abstracted from the arm, and leeches applied to the abdomen: the patient had a slight evacuation, and felt somewhat relieved; the symptoms, however, were not removed, and he was greatly reduced.

On the eighth day, ℥ss. Ol. Ricini was prescribed, which procured four or five copious evacuations from the bowels; the patient, in consequence, felt great relief, the pain and tenderness of the abdomen shortly after subsided, and he gradually recovered.

*Cataract.* A young woman, æt. twenty, was admitted with capsular cataract affecting both eyes, so as to occasion complete blindness, which had existed upwards of two years; the patient was, however, able to distinguish a strong light from darkness. The operation of couching was performed on the left eye, from which no apparent benefit was derived, and she left the hospital. Shortly afterwards, she became better able to distinguish the degrees of light or darkness; in a few days, she obtained a confused view of objects, and ultimately was able to see particular objects pretty clearly. It appears that the retina had been so long inactive in this case, that some time was required for regaining its sensibility.

*Contracted Cicatrix.* A boy, æt. fourteen, was admitted to the hospital, having permanent semiflexion of the fore-arm, in consequence of contraction of the cicatrix from a burn: the contraction had existed ten years. The whole of the cicatrix was excised with a scalpel; the edges of the wound retained in contact by adhesive plaster; and a splint fixed along the anterior part of the limb, which was thus kept in a state of extension during the cure. The wound was healed, and the patient had regained free motion of the fore-arm at the expiration of three months.

*Strangulated Femoral Hernia.* In this patient, a woman, æt. forty, the strangulation had existed twenty hours; during which time bleeding, the warm bath, and fomentations, were employed, without relief to the pain, vomiting, and hiccough, which symptoms had gone on increasing. On her admission, the operation was performed, and the intestine reduced with little difficulty: after the operation, she was bled; and an emollient enema injected, which occasioned free evacuation of the bowels. The inflammatory symptoms were slight: the bleeding was, however, repeated twice; and on the fourth day was ordered ℥ss. Ol. Ricini. The patient left the hospital in three weeks.

*Strangulated Inguinal Hernia.* A man, æt. fifty-six, was received into the hospital with strangulated inguinal hernia: the strangulation had existed twenty-four hours. The operation was immediately performed. On opening the hernial sac, a quantity of bloody serum escaped, and a portion of small intestine presented itself, in a high state of inflammation. After dividing the stricture, the intestine was reduced, although with some difficulty. Severe inflammatory symptoms came on, which were relieved by energetic depletion; (was bled five times in thirty-six hours.) No alvine evacuation having taken place on the third day from the operation, notwithstanding enemata had been administered, he was ordered an oleaginous laxative, which procured copious stools, to his great benefit. Some days after, however, fœcal matter passed out from the wound, and the patient had a recurrence of the inflammatory symptoms: these were relieved by appropriate measures, but the patient having eaten some indigestible food, the inflammation returned, and occasioned his death.

*Recto-vesical Lithotomy.* The patient was sixty-one years of age, and has been subject to symptoms of calculus in the bladder for twenty-five years: the prostate gland was considerably enlarged, and he passed his urine with great pain and difficulty; the urine was loaded with mucus, and occasionally mixed with blood. After a few days' rest, the recto-vesical operation was performed, and two large calculi extracted: after the operation, antiphlogistic means were employed, and the patient enjoyed during some days a state of greater ease than he had experienced for years. On the sixth day after the operation, the urine did not pass away so freely; he was attacked by obstinate rigors, tympanitic abdomen, and died on the eighth day. On examination *post mortem*, extensive sloughing was found in the cellular membrane between the rectum and bladder, where a quantity of urine was collected.

*Gunshot Wound.* A man, æt. twenty-one, was shot in the hand, which occasioned such extensive injury of the parts as to demand amputation: this operation was performed at the wrist joint. The stump, however, did not heal, sloughing of the tendons took place, and the patient died at the expiration of six weeks. After death, an abscess was discovered, extending from beneath the deltoid muscle to some distance beneath the pectoralis major: a collection of serum had taken place in the thoracic cavity of the opposite side.

### PISA.

Pisa, though a large city, contains but 20,000 inhabitants: it is situated on a plain, bounded on the north and east by the Appenines, and on the west by the sea. Pulmonary inflammations, although frequent among the inhabitants, are less so than at Florence; intermittents are not of frequent occurrence since the drainage of the surrounding country. The courses of the university are attended by about 500 students annually; not more than one third of these, however, follow the study of the medical sciences. VACCA was formerly professor of surgery to the university.

A branch from the Societa della Misericordia are here engaged in their charitable offices, and obstetric establishments are established in various quarters of the city. The Spedale di Santa Chiara contains about 300 beds; the wards are airy and clean. Clinical lectures are delivered to the students of the university.

As illustrating the practice employed by Vacca, I here insert two or three cases treated by him, from a work published by one of his pupils.*

*Supposed Hernia.* A woman, æt. forty-three, who had been some time in the hospital Santa Chiara, for some chronic complaint, was suddenly attacked by acute pain in the lower part of the abdomen, which was tense and tender to pressure, vomiting, and hiccough; the pulse was small and frequent. The patient had had an alvine evacuation a few hours before. She stated that she had had hernia some years ago, which was reduced, and she wore a truss for some time, but discontinued its use, and had not since felt any inconvenience. On examining the groin, a tumor was perceived beneath Poupart's ligament, of the size of a large nut: the case was considered by Professor VACCA to be strangulated femoral hernia; yet the tumor not being very tender to the touch, and the patient having been confined to bed for some days previous to the attack, occasioned some doubt. An incision was, however, made though the skin and fascia, and the tumor exposed: an opening was made into the supposed herniary sac, and a small quantity of serum escaped. No intestine or omentum were, however, visible, and the finger could not be passed beyond Poupart's ligament. It was then ascertained that the tumor had been formed of an old herniary sac, which had become closed at its neck, probably from the pres-

* Espositione delle Malattie trattate, dal Professore VACCA: Pisa.

sure of the truss. Antiphlogistic means were employed, but the symptoms increased in intensity, and the patient died twenty-four hours after the operation.

On examining the body, a quantity of extravasated blood was formed in the abdomen, partly in a fluid state, and partly in coagula, which adhered to the surface of the intestines. It was supposed that this proceeded from the rupture of a large vessel; but, on removing it, a fresh collection took place, and it was perceived to transude from the whole of the surface of the intestines, and the peritoneum lining the abdominal parietes; these parts were highly inflamed and thickened. No evidence of intussusceptio, or strangulation of any part of the intestines existed.

*Vertebral Disease.* A woman, æt. twenty-six, had been subject, during some months, to fixed pain in the back, attended with loss of power over her inferior extremities: when admitted to the hospital, the paralysis had so far increased, that she could only move the legs with difficulty: the spinous processes of the eleventh and twelfth dorsal vertebræ projected considerably. Professor Vacca ordered the moxa to be applied on one side of the projection, and the wound kept in a suppurating state. Before the separation of the eschar, an evident amelioration had taken place; the patient was able to move her legs more freely. At the expiration of two months she could walk pretty well, without feeling pain in the back; the spinous projection also appears lessened. At this time the patient complained of pain extending over the right side of the abdomen, she was also feverish towards the evening. On careful examination, a deep-seated tumor was felt, giving a sensation of obscure fluctuation in the right iliac region. After some weeks, the tumor had increased in size, with evidence of fluctuation; the patient had still slight hectic fever; however, she acquired more strength in the inferior extremities, and could walk with ease. No further change took place in the tumor in the course of the two following months; the patient had in the mean time acquired flesh and strength, and left the hospital, where she had been five months.

The opinion of Professor Vacca was that a cure of the caries of the spine had taken place; that a condensation of the matter of the abscess, with adhesion to its cyst, had occurred.

Three months after having left, the patient came to shew herself at the hospital; she had become much fatter, and could walk several miles without inconvenience; the tumor was stationary, having neither increased nor diminished.

Several months after her last appearance, she returned to the hospital in a different state: she stated that the pain in the back returned, accompanied with torpidity of the right thigh. The tumor had increased considerably in size, and another fluctuating tumor was perceptible in the situation of the ischiatic notch; the thigh and leg were œdematous. The patient was still able to walk, and did not enter the hospital: she returned, however, in five weeks, and was admitted; in addition to the two previous tumors, another in the superior part of the thigh had supervened, and pressing the matter from one, increased the size of the others. The termination of this case is not given, but the result may be imagined.

It may be questioned whether the benefit derived in the first instance is attributable to the good effects of the moxa, or to the matter having shifted its situation, and consequently affecting less, by its pressure, the nerves which supply the lower extremities.

*Recto-vesical Lithotomy.* A young man, æt. twenty, was received into the hospital with stone in the bladder; the recto-vesical operation was performed by Professor Vacca in his usual manner: the calculus was seized with facility; but, being large and friable, was broken in attempting to extract it. The portions were extracted singly, and the bladder washed out by injection of tepid water. No serious symptoms followed the operation until the fourteenth day, when the patient felt pain about the neck of the bladder, attended with a frequent desire to void his urine. The symptoms led to the suspicion that a fragment of the calculus remained in the bladder, which was verified by the introduction of a sound. A pair of forceps, was passed with some difficulty through the wound into the bladder, and a large irregular-shaped fragment, which occasioned much pain in its removal, was extracted. A dose of opium was given to the patient after the operation; he was bled, and fomentations applied to the abdomen; fever, however, came on, accompanied with pain and tension of the abdomen. These symptoms were relieved by the means employed: but, three days aftewards, flatus and fluid stercoraceous matter passed away by the urethra each time he had a stool; he was harassed by frequent diarrhœa and tenesmus, and greatly reduced in strength. An elastic gum catheter was passed by the urethra into the bladder: this prevented the entrance of fœcal matter into the bladder, but was obliged to be withdrawn shortly afterwards, in consequence of the increased irritation it occasioned. The diarrhœa at length ceased; the patient regained flesh and strength, and was able to walk about two months after the operation: the fistulous opening, however, remained, through which a great proportion of the urine passed, occasioning much tenesmus. He was about to leave the hospital when he was suddenly attacked by nephritic colic, which was relieved by antiphlogistic means. This attack occasioned suspicion of the presence of a calculus in the pelvis of the kidney; but, as the patient continued pretty well in health, he left the hospital.

Soon after his return home, he experienced fresh symptoms of stone in the bladder; these continued to increase in severity, and obliged him to return to the hospital, after seven months' absence. A calculus was detected by sounding; the fistulous communication between the bladder and rectum remained in the same state. Rest, and the occasional use of the warm bath, were employed for some time. Six weeks after his admission, being considered in a fit state to undergo the operation, the fistulous aperture was enlarged with a bistoury, and a calculus of the size of a walnut extracted; the bladder was then washed out by tepid injection, charpie introduced between the edges of the recent wound, and the patient was bled. Some fever and pain in the hypogastric region having supervened towards the evening, leeches and fomentations were applied, which relieved these symptoms. The wound was healed on the twenty-sixth day from the operation; the patient enjoyed good general health, and was shortly after discharged; the original fistula remaining in the same state.

(To be concluded in our next.)

